# Does the superior fronto-occipital fascicle exist in the human brain? Fiber dissection and brain functional mapping in 90 patients with gliomas

**DOI:** 10.1016/j.nicl.2020.102192

**Published:** 2020-01-22

**Authors:** Xiaoliang Liu, Masashi Kinoshita, Harumichi Shinohara, Osamu Hori, Noriyuki Ozaki, Mitsutoshi Nakada

**Affiliations:** a Department of Neurosurgery, Kanazawa University,13-1 Takara-machi, Kanazawa, 920-8641 Japan; b Department of Functional anatomy, Kanazawa University, Japan; c Department of Neuroanatomy, Kanazawa University, Japan; d Department of Neurosurgery, The First Hospital of Jilin University, China

**Keywords:** Acallosal Brain, Awake Surgery, Fiber Dissection, Superior Fronto-occipital Fascicle, Voxel-based Morphometry Analysis

## Abstract

The presence of the superior fronto-occipital fascicle (SFOF) has been reported in the Rhesus monkey; however, it is a subject of controversy in humans. The aim of this study is to identify the SFOF using both *in vitro* and *in vivo* anatomo-functional analyses.

This study consisted of two approaches. First, one acallosal brain and 12 normal postmortem hemispheres (five left and seven right sides) were dissected under a microscope using Klingler's fiber dissection technique. We focused on the medial subcallosal area superior to the Muratoff bundle, which has been indicated as a principal target area of the SFOF in previous studies. Second, 90 patients underwent awake craniotomy for gliomas with direct electrical stimulations. Functional examinations for visual, ataxic, and cognitive tasks were performed and 453 positive mapping sites were investigated by voxel-based morphometry analysis to establish the functions of the SFOF.

The corticostriatal fibers, or the Muratoff bundle, and thalamic peduncle fibers joined in the area of the caudate nucleus, making thalamic peduncle/ corticostriatal bundles, which ran antero-posteriorly in the anterior subcallosal area and radiated from the caudate superior margin in the posterior subcallosal area. However, no SFOF fiber bundle crossed perpendicular to the thalamic peduncle/ corticostriatal bundles in the posterior subcallosal area. In the acallosal hemispheres, Probst bundles were confirmed and the subcallosal areas did not show a specific organization different from the normal brain. Hence, we could not detect a long and continuous association fascicle connecting the frontal lobe and occipital or parietal lobe in the target areas. Furthermore, in the *in vivo* functional mappings of awake surgery and voxel-based morphometry analysis, eight positive points on the SFOF were selected from the total 453 positive points, but their functions were not related with visual processing and spatial awareness, as has been reported in previous studies.

In conclusion, in the present study we attempted to investigate the existence of the SFOF using an anatomical and functional approach. According to our results, the SFOF may not exist in the human brain.

## Highlights

• Existence of superior fronto-occipital fascicle (SFOF) in humans is controversial.
• Fiber dissection in vitro revealed Muratoff and Probst bundles but not SFOF.
• Direct functional mappings for SFOF were performed in 90 awake craniotomies.
• Eight of total 453 positive sites were located in the region believed to be SFOF.
• The anatomo-functional features suggest that SFOF might not exist in human brain.

## 1 Introduction

In 1895, Dejerine delineated the original location and trajectory of the superior fronto-occipital fascicle (SFOF) when performing post-mortem dissections on the normal human brain (Dejerine, 1895). Since then, the prevailing consensus is that the SFOF, connecting the frontal lobe and occipital/parietal lobe, is an association fiber tract 2-3 mm in diameter, just under the ependyma, superolateral to the body and head of the caudate nucleus, medial to the corona radiata, and inferolateral to the corpus callosum fibers. Schmahmann and Pandya later identified the location and trajectory of the SFOF in their isotope axonal tracing studies on the monkey brain (Schmahmann and Pandya, 2006; Schmahmann and Pandya, 2007). The SFOF is situated above the Muratoff bundle, lateral to the corpus callosum, and medial to the corona radiata. However, Türe and Yasargil failed to reveal an association fiber tract in this region in the normal human brain using Klingler's fiber dissection technique (Türe et al., 1997). Diffusion tensor image (DTI) tractography is a recently developed technique that can visualize white matter fibers by measuring the anisotropic nature of water diffusion along myelinated axon fibers (Potgieser et al., 2014). It has been used extensively to reveal white matter fibers *in vivo* (Catani et al., 2002; Hagmann et al., 2003) and provides a powerful tool to demonstrate finer changes in white matter fibers (Tovar-Moll et al., 2007). Using this technique, some studies have delineated the SFOF in the human brain (Catani et al., 2002; Wakana et al., 2004; Makris et al., 2007). However, a recent study using more advanced fiber tractographies could not reveal its existence, location, and trajectory in the normal human brain (Forkel et al., 2014; Meola et al., 2015; Mori et al., 2002; Milian et al., 2014).

In 1887, an important thesis by Onufrowicz reviewed 27 single-case reports of acallosal brains using gross dissection and described their anatomic features, including an association system, the “SFOF”, connecting the frontal and occipital lobes (Onufrowicz, 1887; Schmahmann and Pandya, 2006; Forkel et al., 2014). Although Onufrowicz and subsequent researchers impelled the understanding of the anatomy of the acallosal brain, the hand-painted or schematic drawings of the anatomic configurations could not depict white matter fibers. The acallosal brain is a very complex condition which requires multidisciplinary analyses, including fiber dissection.

The potential functions of the SFOF, such as immediate top-down control of visual processing and spatial awareness, have been reported (Karnath et al., 2009; Schmahmann and Pandya, 2006; Thiebaut de Schotten et al., 2011). Today, awake craniotomy allows neurosurgeons to monitor eloquent brain functions in real-time during brain surgery for tumors such as gliomas (Dziedzic and Bernstein, 2014; Gras-Combe et al., 2012; Milian et al., 2014). To date, there is no report regarding intraoperative functional assessments of the SFOF in humans. To resolve the anatomo-functional controversy regarding the SFOF, we first performed *in vitro* Klingler's fiber dissection of postmortem cerebral hemispheres including the acallosal brain, and *in vivo* electrophysiological analysis using direct electrical stimulation (DES) during awake craniotomy on 90 patients with gliomas. Finally, the affected brain regions were evaluated with voxel-based morphometry (VBM) analysis to confirm the exact functions of the SFOF, if it existed, or which fascicles carry the assumed functions of the SFOF in the human brain.

## 2 Materials and methods

### 2.1 Fiber dissection technique

Fiber dissection was performed on one acallosal brain and 12 normal postmortem hemispheres (five left and seven right sides). The cadavers were voluntarily donated to Kanazawa University School of medicine for research and educational purposes. They were perfused with 4.4% formalin injected into a femoral artery and blood was drained from the ipsilateral femoral vein and bilaterally from the internal jugular veins. One week later, the brains were isolated from the body and kept in 5% formalin for at least 3 months. After removing the arachnoid and blood vessels, the brains were subjected to Klingler's freezing-thawing method at least twice. Fiber dissections for cadavers were approved by the ethics committee at Kanazawa University [No.2018-201(2956)].

Bamboo spatulas were used for crude removal of the gray and white matter (Supplementary Fig. 1). Dissection to expose fiber bundles in the white matter for electron microscopy was carried out manually with fine-tipped forceps. The trajectory of a fiber fascicle was pursued and confirmed primarily by peeling off minute fiber fractions. Microdissection was performed under a stereoscopic microscope (Olympus MXZ 7). Needles attached to colored balls were used as landmarks.

### 2.2 Patients

Ninety patients with gliomas participated in this study. They underwent awake craniotomies at Kanazawa University Hospital from May 2014 to January 2019. Patient data is shown in Table 1. Written informed consent for the use of images and neuropsychological data was obtained from all patients in the study, and all data were collected retrospectively from their medical records. The study was performed in accordance to the guidelines of the institutional review board of Kanazawa University [No. 2017-151 (2593)].

**Table 1:** Patients characteristics (n = 90)

	Number
Mean age (y.o.) [range]	47.6 [14-73]
Gender	
Male	60 (67%)
Female	30 (33%)
Location	
Left	49 (54%)
Right	41 (46%)
Lobe	
Frontal	43 (48%)
Temporal	27 (30%)
Parietal	16 (18%)
Occipital	4 (4%)
WHO grade	
I	2 (2%)
II	27 (30%)
III	31 (34%)
IV	30 (33%)

### 2.3 Brain functional mapping and VBM analysis

All surgical procedures were performed using an asleep-awake-asleep technique along with a direct stimulation mapping by the same operator (M.K.) (Duffau et al., 2008; Szelenyi et al., 2010). After dural incision, brain functional mapping (cortical and subcortical mapping) was evaluated and preserved, with DES delivered via a bipolar probe with a 5-mm space between the tips that delivered a biphasic current (pulse frequency 60 Hz, single-pulse phase duration 0.2 ms and amplitude 2-6 mA). During awake surgery, brain functional mapping tasks, including a counting test for speech function; picture naming test, pyramids and palm trees tests for language function; motor, sensory, and line bisection tests for visuospatial and visual ataxic functions (Thiebaut de Schotten et al., 2005); spatial 2-back test for spatial working memory (Kinoshita et al., 2016); emotion and theory of mind tests for socio-cognitive function (Nakajima et al., 2018); and calculation, reading, and 4-screen picture naming tests for visual field (Gras-Combe et al., 2012) were performed. We subsequently recorded each symptom and confirmed the volume of the positive mapping point according to the correspondence correlation between intensity of the stimulation current and the depth of the stimulation point in lower thresholds of DES with no more than 6 mA (Shiban et al., 2015) (Supplementary Fig. 2). All positive mapping sites were recorded in intraoperative pictures and videos and were reconfirmed by a neuro-navigation system (Curve system and iPlan3.0 software, BrainLab).

Structural magnetic resonance (MR) images were acquired during the 2-3 months postoperative period. The lesion resection cavities of 90 patients were reconstructed in standardized Montreal Neurological Institute (MNI152) space (resolution of 1 × 1 × 1 mm) using Statistical Parametric Mapping (SPM) 12 implemented in a MATLAB environment (R2018b, version 9.5; The MathWorks, Inc.) with cost function masking (Brett et al., 2001) and MRIcron software (Chris Rorden, MRIcron 2016, https://www.nitrc.org/projects/mricron/). The reconstructed volume of interest (VOI) was compared with a non-normalized image and intraoperative records. All the positive points were overlapped in a brain template. We then analyzed the positive points on the SFOF region which was reconstructed by Diffusion Spectrum Imaging (DSI) Studio (freely downloaded at: http://dsi-studio.labsolver.org/) in the human connectome project (HCP)-842 Template (2015 Q3, 900-subject release. Downloaded at: http://dsi-studio.labsolver.org/download-images/hcp-842-template).

### 2.4 Parameters for Diffusion Spectrum Imaging (DSI) tractography

Fiber tracking was initiated separately for each orientation and fiber progression continued with a step size of 1.2 mm, minimum fiber length of 20 mm, and turning angle threshold of 60°. If multiple fiber orientations existed in the progression location, the fiber orientation that was nearest to the incoming direction and formed a turning angle smaller than 60° was selected to determine the next moving direction (Fernandez-Miranda et al., 2015). To smooth each track, the next moving directional estimate of each voxel was weighted by 20% of the previous incoming direction and 80% of the nearest fiber orientation. This progression was repeated until the quantitative anisotropy of the fiber orientation dropped below a preset threshold (0.03–0.06 depending on the subject) or there was no fiber selected within the 60° angular range of progression (Yeh et al., 2013). The SFOF and other related fascicles including the pyramidal tract, somatosensory tract, arcuate fascicle, and inferior fronto-occipital fascicle were reconstructed by referring to previous studies (Fernandez-Miranda et al., 2015; Meola et al., 2015; Wu et al., 2016) (Supplementary Fig. 3).

## 3 Results

### 3.1 Fiber dissection in the normal brain

**Fig. 1:**
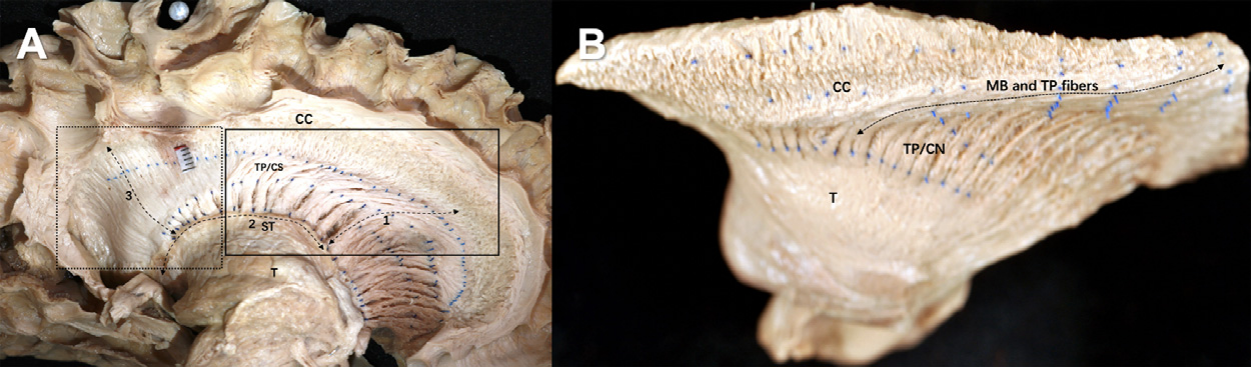
**Fiber dissection of the normal brain. (A)** Medial approach of the fiber dissection in the normal brain. A paper scale with 5 mm was put in the brain. The ball indicated the central sulcus. The solid line square indicated an anterior part of the subcallosal area and the dotted line square indicated a posterior part of the subcallosal area. The Muratoff bundle (MB) and thalamus peduncles (TP) fibers ran anteroposteriorly in the solid line square, while the MB, TP, and corpus callosum (CC) fibers ran in a near supero-inferior orientation in the dotted line square. Blue needles with 0.3 mm in diameter were used. Three needles were inserted at each root of TP/ corticostriatal (CS) fibers, at the superior border of the caudate nucleus, and at the corpus callosal-subcallosal area border. Thus, three rows of needles were arranged in an antero-posterior direction. Additionally, the fourth needle was inserted between the first and second row of needles. The tag 1 and tag 3 indicated the TP/CS. These TP/CS fibers seemed to connect with the tag 2 (stria terminalis, ST). CC = corpus callosum, MB = Muratoff bundle, TP = thalamus peduncles, CS = corticostriate, ST = stria terminalis. **(B)** Superior approach of the fiber dissection in the normal brain. The MB and TP fibers (arrow) coursed above the body and head of the caudate nucleus (CN) in an anteroposterior direction, and descended into the CN. Blue needles were put along the bundle of fibers. CC = corpus callosum, MB = Muratoff bundle, TP = thalamus peduncles, T = thalamus, CN = caudate nucleus.

The medial approach is the shortest and most direct way to get to the location of the SFOF. Blue needles 0.3 mm in diameter were placed along the fibers. The corticostriatal fibers or Muratoff bundles, and the thalamic peduncle fibers joined in the area of the caudate nucleus, making thalamic peduncle/ corticostriatal bundles. In the anterior part of the subcallosal area (Fig. 1A, solid line square), thalamic peduncle/ corticostriatal fibers presented an anteroposterior orientation at the level of the superolateral margin of the caudate and reached downward to the thalamus (Fig. 1A tag 1). In the posterior part of the subcallosal area (Fig. 1A, dotted line square), retral thalamic peduncle/corticostriatal fibers presented a supero-inferior orientation and reached downward to the thalamus (Fig. 1A tag 3). Hence, if the SFOF, which is made of horizontal association fibers connecting the frontal and occipital lobes, exists it would be clearly discriminated within the dotted square. Therefore, we deemed fiber dissection to be an appropriate technique to investigate the SFOF. Both antrorse and retral thalamic peduncle/ corticostriatal bundles were adjacent to the stria terminalis (Fig. 1A tag 2), which passes through glial tissue along with the thalamostriate blood vessels. In the subcallosal area, we failed to confirm any continuous fascicle connecting the frontal lobe and occipital or parietal lobe in anteroposterior direction.

Considering that the SFOF is located above the Muratoff bundle, which separates it from the caudate nucleus, we used the following approach to reveal the SFOF. After removing the cingulate gyrus and corpus callosum, the trajectories of the fiber fascicle were pursued and confirmed by peeling off antero-posteriorly. In our fiber dissection, the Muratoff bundle fibers intermingled with the thalamic peduncle fibers and coursed above the body and head of caudate nucleus in an anteroposterior direction, descending into the striatum (Fig. 1B, arrow). However, no anteroposterior fascicle that was compatible with the SFOF was found in the stepwise fiber dissection.

### 3.2 Fiber dissection in the acallosal brain

**Fig. 2:**
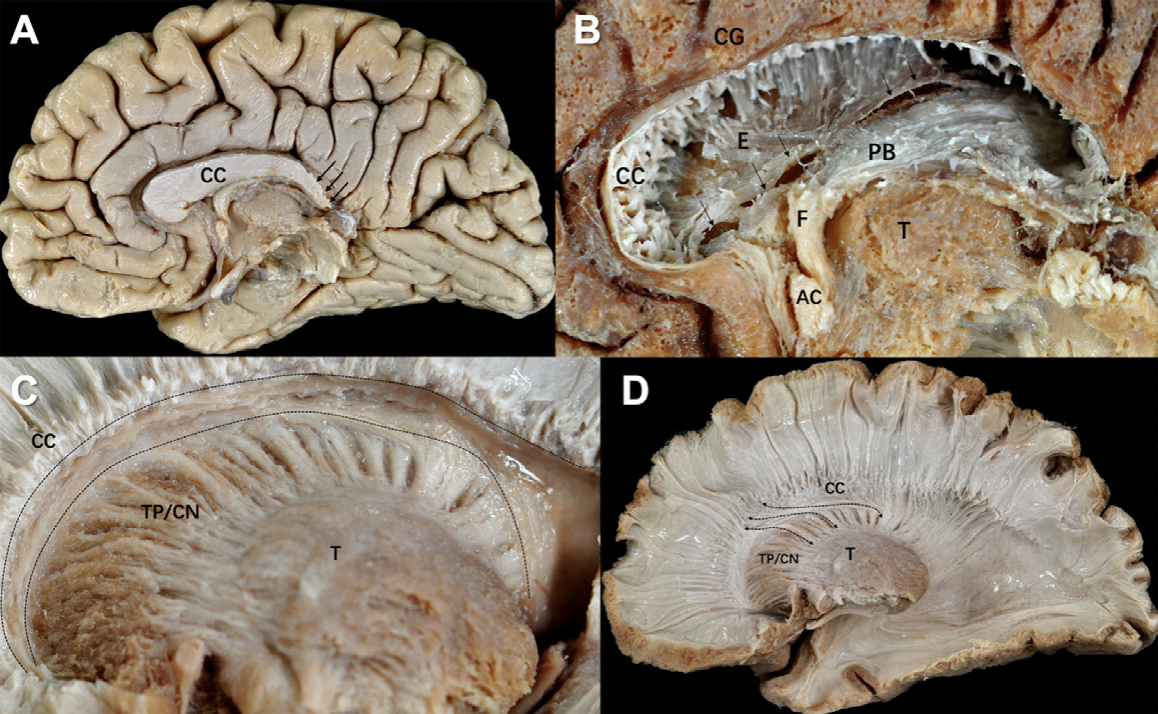
**Fiber dissection of the acallosal brain. (A)** The medial surface of the acallosal brain. We could not discriminate the beak of the corpus callosum (CC). CC had a relatively thickened anterior part, while the posterior part was attenuated (arrows). The splenium was absent. CC = corpus callosum. **(B)** In the acallosal hemisphere, the thickened anterior corpus callosum was a mass of white matter consisting of variously oriented fibers. Thus, this mass is compatible with the Probst bundle (PB). It pushed down the ependyma and crus of the fornix to the thalamic surface. The ependyma was discontinuous (arrows) along the lamina affixa to which the choroid plexus attached. The PB fibers and fornix made a mass of fibers, which was large enough to bury almost the entire anterior horn of the lateral ventricle (PB/F). CC = corpus callosum, E = ependyma, T = thalamus, F = fornix, AC = anterior commissure, PB = Probst bundle, CG = cingulate gyrus. **(C)** The subcallosal area was indicated by the stippled lines in the acallosal brain. The area was located between the inferior surface of the corpus callosum (CC) and the superior margin of the caudate nucleus (CN). Although the subcallosal areas in the acallosal brain were rather narrow, they were within the normal variations and did not show a different organization from the control fibers. The thalamic peduncles originated from the thalamus, ran through the caudate nucleus and curved acutely to anterior at the superior boarder of the caudate nucleus. The posterior peduncle bundle flanked medially to the anterior bundle and, thus, a narrow ceiling of the caudate nucleus was made by the peduncular bundles. This ceiling was triangular in a coronal view and measured approximately 3 mm base x 3 mm height. CC = corpus callosum, CN = caudate nucleus, T = thalamus, TP = thalamic peduncles. **(D)** After exposing the total subcallosal areas, only antrorse TP fibers (arrows) in the anteroposterior direction could be found. And removing the corpus callosum (CC) to expose the area above the subcallosal area, no association fibers could be found. CC = corpus callosum, CN = caudate nucleus, T = thalamus, TP = thalamic peduncles.

The medial surface of the acallosal brain presented with an attenuated posterior half of the corpus callosum and absence of splenium (Fig. 2A, arrows). The corpus callosum and fornix, made up of a mass of fibers with a variety of orientations, was large enough to bury almost the entire anterior horn of the lateral ventricle (Fig. 2A). The mass was removed first by dividing it into small fiber bundles which were subsequently pinched off with forceps. The direction of the fibers was not always rectangular to the sagittal plane and tended to change from antero-medial to postero-lateral, especially near the fornix. This mass of fibers (Fig. 2B, PB) rested on a thin layer of the ependyma (Fig 2B, E). The ependymal layer was discontinuous along the lamina affixa on the thalamus (Fig. 2B, arrows), which appeared to arbitrarily divide into the antero-superior callosal and postero-inferior fornix fiber areas. After removing the caudate and ependyma and cutting half the corpus callosum, the subcallosal areas was exposed. The subcallosal areas (Fig. 2C, the stippled lines) of the acallosal brain were narrow, but were within the normal variations and did not show an organization different from the normal brain. After exposing the total subcallosal areas, only antrorse thalamic peduncle fibers in the anteroposterior direction could be found (Fig. 2D, arrows). Removing the corpus callosum to expose the area above the subcallosal area in a stepwise fashion did not reveal any association fibers similar to those mentioned in the monkey brain. A full description is given in Supplementary material 1.

### 3.3 Brain functional mapping and voxel-based morphometry analysis

**Fig. 3:**
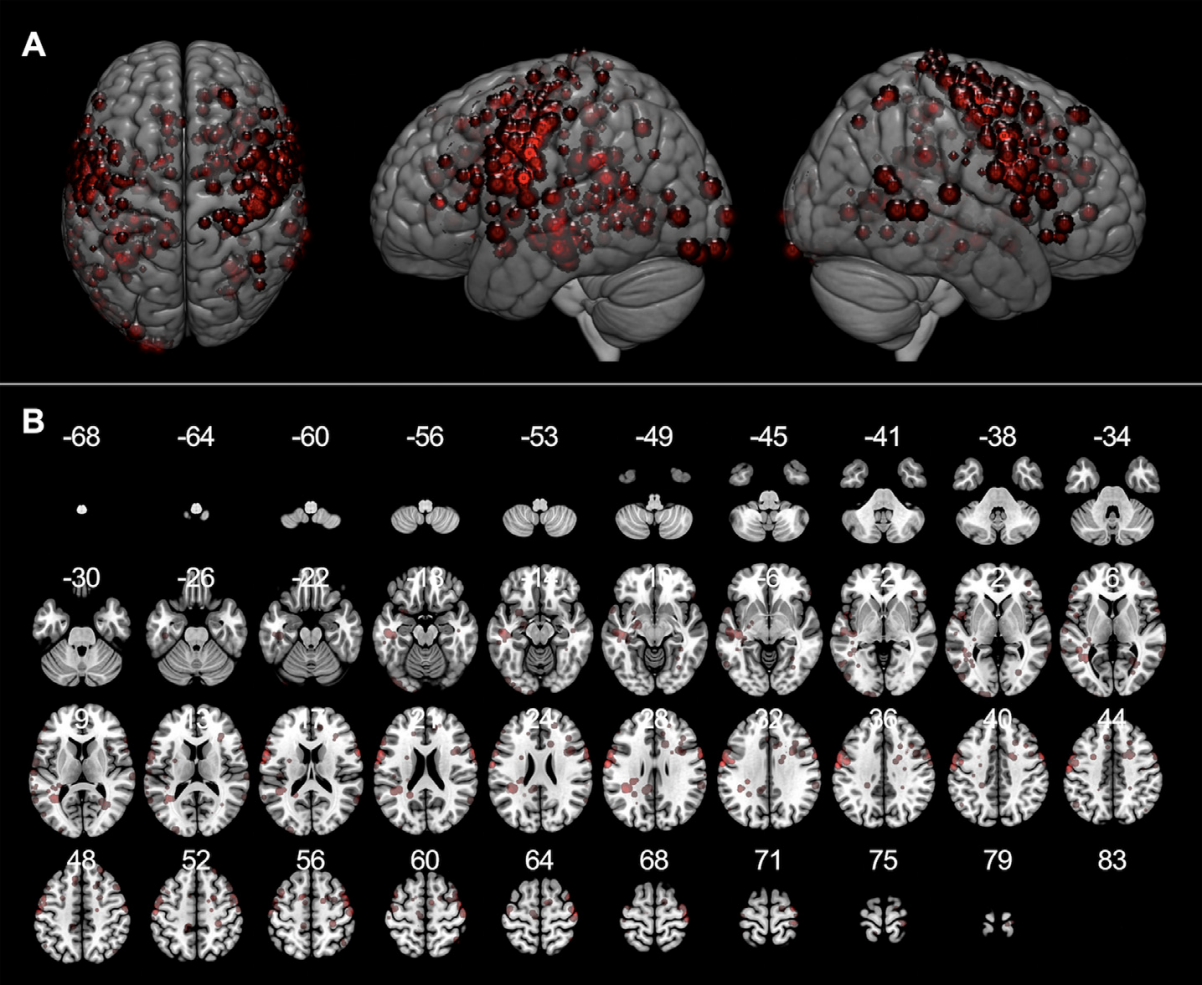
Brain functional mapping and voxel-based morphometry analysis. All 453 positive mapping points present in three-dimensional brain images (A) and axial images (B) with Montreal Neurological Institute (MNI) coordinates.

**Fig. 4:**
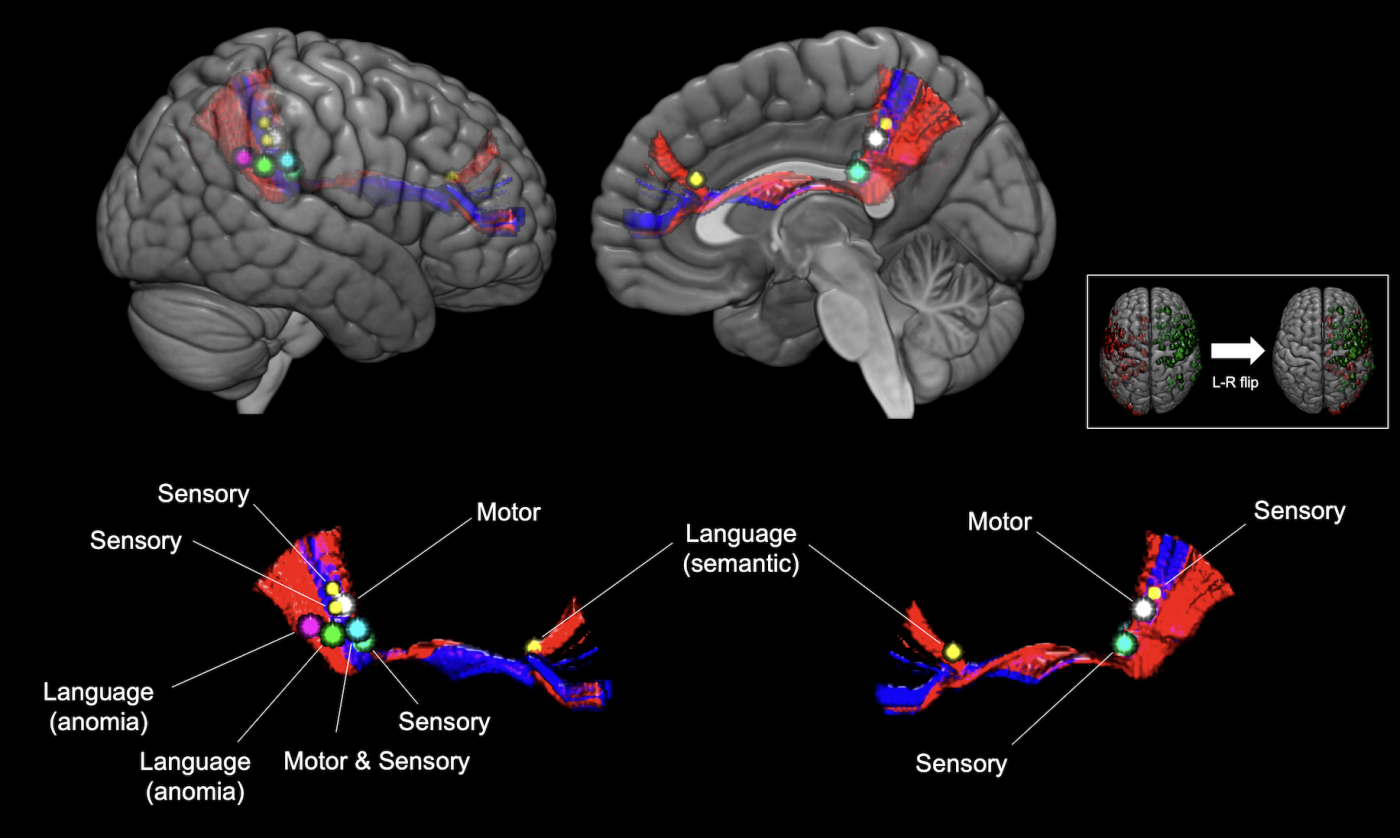
**Three dimensional anatomical images with relationships between superior fronto-occipital fascicle (SFOF) and positive mapping points.** Eight positive mapping points including three language points (magenta, green, and yellow), three sensory points (two yellow and one cyan), one motor point (white), and one both motor and sensory point (cyan) were confirmed on the SFOF. The positive mapping points and SFOF in the left hemisphere are fused into right side shown in small white box. SFOF, superior fronto-occipital fascicle.

**Fig. 5:**
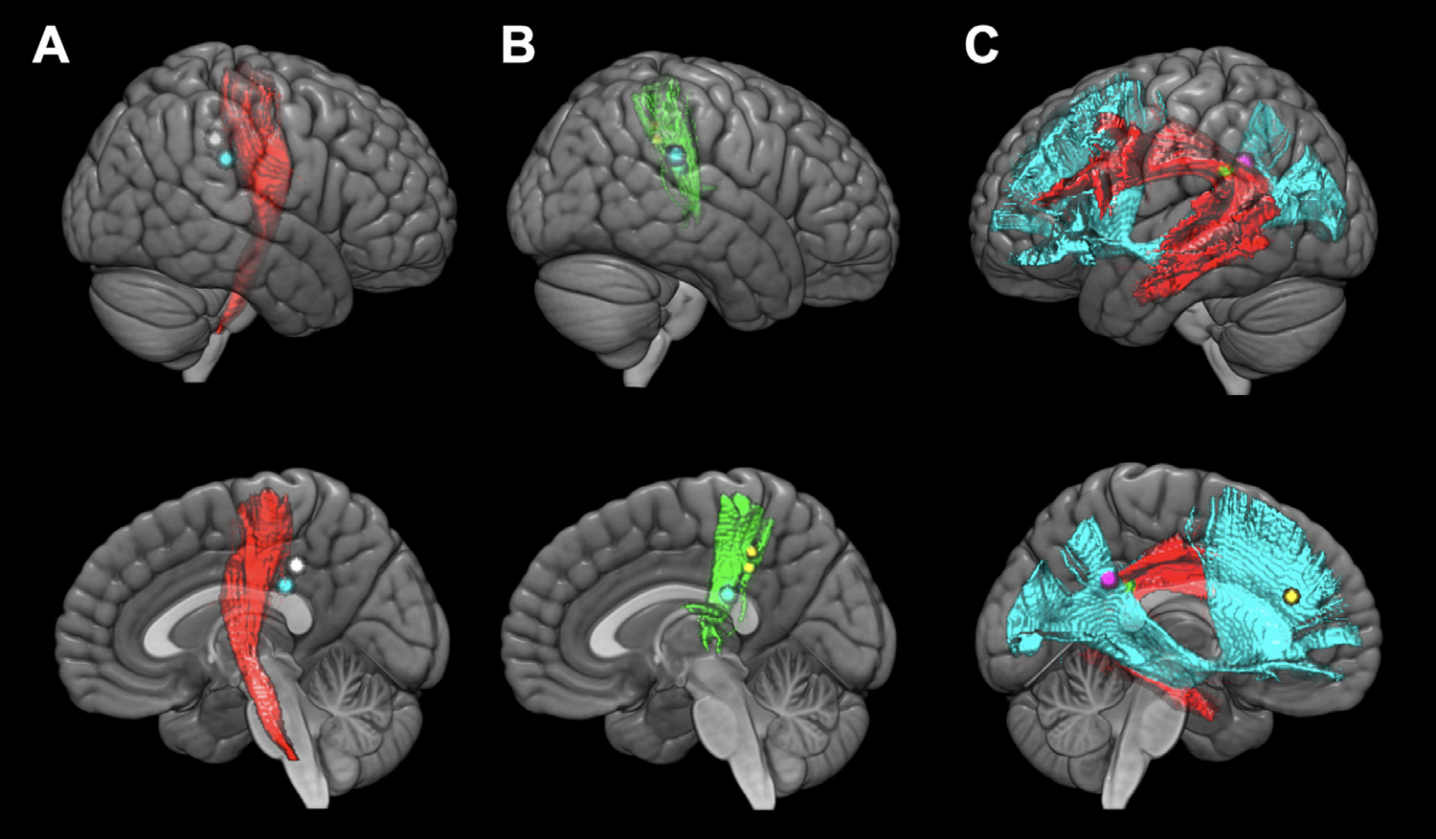
**Structural relationships between white matter tracts and positive mapping points anatomically concordant with the superior fronto-occipital fascicle.** Specific tracts reconstructed by diffusion spectrum imaging were confirmed to contribute to the functions of eight positive mapping points in three-dimensional brain images. Motor positive points (white and cyan) are related with Pyramidal tract (red) (A), and sensory positive points (two yellow and one cyan) are related with somatosensory tract (green) (B). Language positive points (magenta, green, and yellow) are related with arcuate fascicle (red) and inferior fronto-occipital fascicle (cyan) (C). Each upper image is lateral view and lower is medial view.

**Fig. 6:**
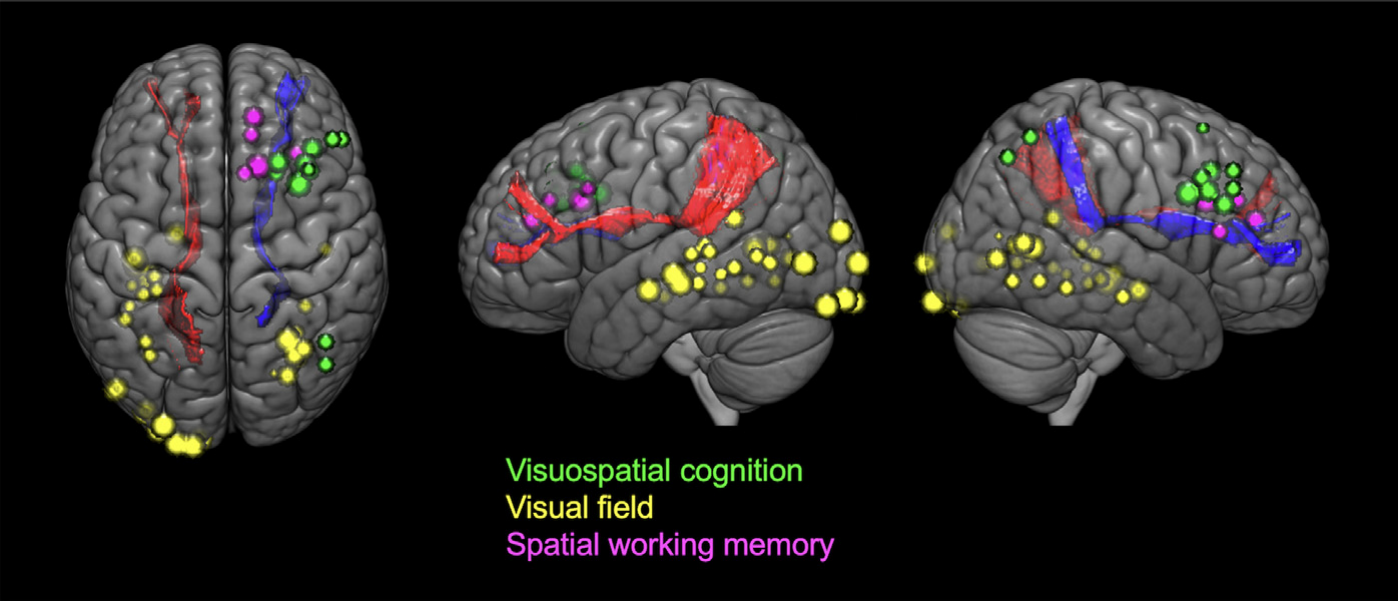
**Structural relationships between white matter tracts and positive mapping sites involved in visual processing and spatial awareness.** The positive mapping points including visuospatial cognition (green balls), visual field (yellow balls) and spatial working memory (pink balls) are overlapped and separated from the superior fronto-occipital fascicles (left, red; right, blue).

We confirmed a total of 453 positive mapping points in the 90 patients (Fig. 3A). Detailed characteristics of these 453 positive mapping points are summarized in Table 2. Eight positive mapping points were selected on the SFOF and all the points underwent left to right flip (L-R flip) (Table 3, Fig. 4, and Supplementary Fig. 4). The positive mapping sites on the SFOF included 3 language points, 3 sensory points, 1 motor point, and 1 combined motor and sensory point. None were symptoms of disconnection of the tract, but of other networks such as the pyramidal tract (Fig. 5A), somatosensory tract (Fig. 5B), and language-related tracts including left arcuate fascicle and left inferior fronto-occipital fascicle (Fig. 5C). Especially in language disorders induced by DES, the distribution of anomia and semantic paraphasia could explain the language networks in detail. The positive mapping points involving visuospatial cognition, visual field, and spatial working memory did not map to the SFOF (Fig. 6). Thus, from the data obtained from intraoperative brain mapping and DSI tractography, we could not identify the functions of the SFOF, which were previously reported to be visual processing and spatial awareness.

**Table 2:** Characteristics of intraoperative positive mapping points

Induced symptoms	Number (%)
Speech disorder	123 (27.2%)
Anarthria	64
Dysarthria	24
Speech arrest	18
Others	17
Motor disorder	112 (24.7%)
Involuntary movement	65
Motor control disorder	47
Language disorder	73 (16.1%)
Anomia	23
Phonemic paraphasia	13
Semantic paraphasia	20
Others	17
Sensory disorder	68 (15.0%)
Visual field disorder	30 (6.6%)
Socio-cognitive disorder	27 (6.0%)
Visuospatial disorder	19 (4.2%)
Others	20
Total	**453**
Threshold of stimulation	
1.5--2.0 mA	3 (3%)
2.5--3.0 mA	22 (24%)
3.5--4.0 mA	35 (39%)
4.5--5.0 mA	19 (21%)
5.5--6.0 mA	11 (12%)
Mean 4.0 (±1.1) mA

**Table 3:** Positive mapping points corresponding with the tract of superior fronto-occipital fascicle

Positive mapping point	MNI coordinate (x, y, z)	Threshold of stimulation (mA)
Language 4	(−34, −40, 27))	6
Language 18	(−27, −48, 30)	5
Language 33	(−16, 33, 22)	4
Motor 27 and Sensory 42	(−29, −31, 29)	5
Motor 28	(−22, −36, 38)	5
Sensory 17	(−24, −29, 25)	5
Sensory 29 A	(25, −39, 37)	3
Sensory 29B	(23, −40, 44)	3

## 4 Discussion

In monkeys, the SFOF was identified using isotopic labeling. Later DSI confirmed and isolated the SFOF as a discernible bundle that coursed above the body and head of the caudate nucleus, medial to the corpus callosum, lateral to the corona radiata, and separated from the caudate nucleus by the Muratoff bundle (Schmahmann et al., 2007). However, confusion and intense debate regarding the existence of the SFOF in the human brain persists (Forkel et al., 2014; Schmahmann and Pandya, 2007). Recently, convincing evidence from a DSI study denied the existence of the SFOF in the normal human brain (Meola et al., 2015). The study suggested that the superior thalamus peduncles, stria terminalis, and posterior thalamus peduncles produce the appearance of a continuous association fiber that connects the parietal and frontal lobes.

With our medial approach, we were able to directly expose the assumed location of the SFOF just after removing the ependyma and the caudate nucleus, avoiding complex fiber systems such as the SLF, arcuate fascicle, and internal capsule fibers. Furthermore, the 0.3 mm diameter needles were arranged in an anteroposterior orientation (Fig. 1A). If the SFOF existed, the needles could help us to easily identify it in the anteroposterior orientation. The results of our fiber dissection in normal brains were in general agreement with previously reported DSI tractography. We found that both the antrorse thalamic peduncle and retral thalamic peduncle showed a spurious continuation with the stria terminalis. Fiber dissection allowed us to obtain more detailed information. We observed that the stria terminalis runs in the groove of thalamostriate sulcus with the thalamostriate vein, which curved with the subependymal glial tissue. Overall, it was difficult to find continuous fibers from the cortex to this region using DSI or DTI.

The history of the SFOF is related to the Muratoff bundle and has been well documented in the monkey brain (Schmahmann and Pandya, 2006; Schmahmann et al., 2007). The superior approach provided us a chance to find the Muratoff bundle and resolved the confusion regarding the SFOF. We found that the Muratoff bundle coursed above the head and body of the caudate nucleus in an anteroposterior direction and descended into the striatum (Fig. 1B). It is possible that Dejerine considered that the SFOF was located just under the ependyma because he was unable to distinguish the SFOF from the Muratoff bundle. Thus, according to our fiber dissection analysis, some of the fibers above the caudate nucleus in Dejerine's study of coronal brain sections likely correspond to the Muratoff bundle (Obersteiner and Redlich, 1902).

The Muratoff bundle is composed of corticostriatal fibers that arise from the cortex and lead to the caudate nucleus. Naturally, it accumulates to a high density in the subcallosal area; in the area above this highly dense region, which also corresponds to the SFOF location, the density of the Muratoff bundle becomes similar to that of the SFOF. Thus, at the location of the SFOF, it is impossible to differentiate between the Muratoff bundle and the SFOF. In the anterior part of the subcallosal area, we found that the SFOF ran parallel to the Muratoff bundle and the thalamic peduncle, making it very difficult to differentiate the SFOF from these similar fibers using fiber dissection. However, in the posterior part of the subcallosal area, the Muratoff bundle, thalamic peduncle, and corpus callosum fibers were in a supero-inferior orientation, which differed from the orientation of the SFOF.

At present, few postmortem fiber dissection studies have been performed on acallosal brains, possible because such specimens are scarce. Agenesis of the corpus callosum (ACC) can be classified into three types: complete ACC, partial ACC, and hypoplasia. The acallosal brain represents the former two types (Forkel et al., 2014). Onufrowicz described an association system, the “SFOF”, connecting the frontal and occipital lobes in the acallosal brain. However, Heinrich Sachs (1892) and Moriz Probst (1901) concluded that Onufrowize's supposed SFOF described in acallosal patients was in fact misplaced callosal fibers (the Probst bundle) that had failed to cross to the opposite hemisphere (Probst, 1901; Sachs, 1892). The Probst bundle has been well documented by DTI (Benezit et al., 2015; Lee et al., 2004; Tovar-Moll et al., 2007) and more advanced tractography studies (Forkel et al., 2014). In our fiber dissection, we confirmed the presence of the Probst bundle, which was composed of a mass of fibers in the lateral ventricle. The mass of fibers fusing with the fornix corresponds to Onufrowize's SFOF (Onufrowicz, 1887); thus, our fiber dissection results support the notion that the SFOF observed by Onufrowize's in acallosal patients was the Probst bundle. Interestingly, the fiber organizations in our acallosal brain did not differ from those in the normal brain. These observations will hopefully promote a better understanding of the complexity of acallosal brains in the future.

From a functional viewpoint, awake craniotomy in 90 patients with gliomas did not reveal activity related to visual processing and spatial awareness, which are the assumed functions of the SFOF. Although eight positive points in the total 453 positive points were confirmed at the location of the SFOF, the induced symptoms in the eight positive points have been associated with electrical stimulation of other functional white matter networks. For example, the corticospinal tract and somatosensory tract have been associated with; muscle contraction and sensory disorder, respectively (Sarubbo et al., 2015); the arcuate fascicle with language processing including anomia (Duffau et al., 2002); and the inferior fronto-occipital fascicle with semantic paraphasia (Almairac et al., 2015). Furthermore, other fiber networks, and not the SFOF, were likely involved in tasks for visual processing and spatial awareness. To the best of our knowledge, this is the first human brain functional study using awake craniotomy with direct electrical stimulations to attempt to detect the SFOF. Our results did not support the existence of the SFOF. Further studies for other brain functions must be investigated by awake mapping.

In the context of clinical practice, our findings highlight that the visualization of the SFOF in DTI or DSI might mislead neurosurgeons to excessively preserve the surrounding tissues during surgical resection of tumor. MRI tractography is unable to give detailed insights into white matter pathways because it cannot directly visualize the distinct anatomy of fibers; it only provides an indirect reconstruction based on the diffusion of water molecules (Duffau, 2014; Kinoshita et al., 2015). On the other hand, our results suggest that improvident resection of the SFOF could result in the interruption of other white matter fascicles such as the pyramidal tract, somatosensory tract, arcuate fascicle, and inferior fronto-occipital fascicle. Therefore, not only the SFOF, but also other bundles in tractography should be validated by intraoperative electrophysiological examinations in surgical practice, especially for gliomas.

## 5 Conclusions

In the current study, we investigated the SFOF in terms of anatomy and function. According to our results, the SFOF may not exist in the human brain. Our hope is that this study contributes to solving the controversy of the SFOF in the human brain and promotes further anatomical and functional studies.

## Funding

This work was supported by the China Scholarship Council (National construction of high-quality University projects of graduates to XL); and JSPS KAKENHI (17K10859 and 18H03126 to MK).

## CRediT authorship contribution statement

**Xiaoliang Liu:** Methodology, Investigation, Visualization, Writing - original draft. **Masashi Kinoshita:** Conceptualization, Methodology, Formal analysis, Writing - review & editing, Visualization, Supervision. **Harumichi Shinohara:** Validation, Resources, Data curation, Writing - review & editing. **Osamu Hori:** Validation, Resources, Data curation. **Noriyuki Ozaki:** Validation, Resources, Data curation. **Mitsutoshi Nakada:** Writing - review & editing, Project administration.

## Declaration of Competing Interest

The authors report no competing interests.

## Appendix Supplementary materials

Image, application 1
