# Supplementary material for: Does the superior fronto-occipital fascicle exist in the human brain? Fiber dissection and brain functional mapping in 90 patients with gliomas
Source: Neuroimage Clin. 2020 Jan 22;25:102192. doi: 10.1016/j.nicl.2020.102192 (PMC6997620; doi:10.1016/j.nicl.2020.102192)
Supplement: Supplementary file 1 [file mmc1.pdf]

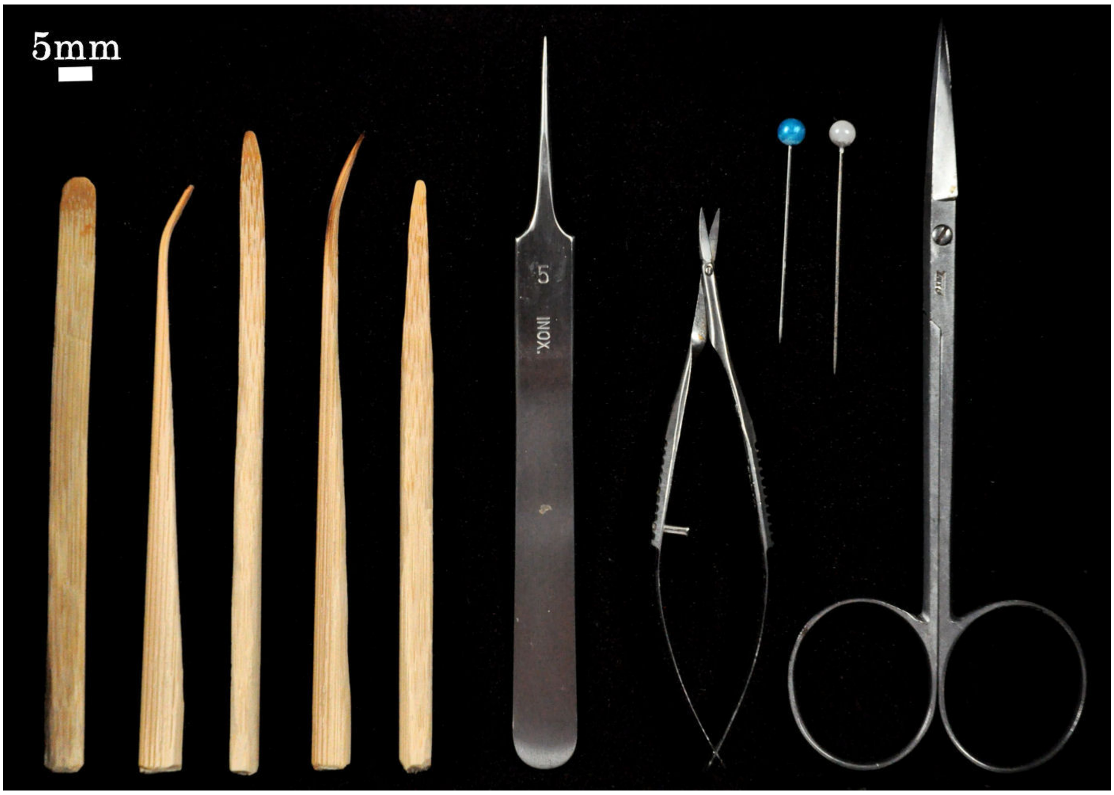

Supplementary Figure 1. Instruments for fiber dissection (explanations from the left).

The instruments for the fiber dissection. Five spatulas made of bamboo, forceps for electron microscopy, an iridectomy scissors, waiting needles with balls, and a medium-sized scissors were shown. The needles were used as a landmark as well as a scale since the ball was 4 mm in diameter.

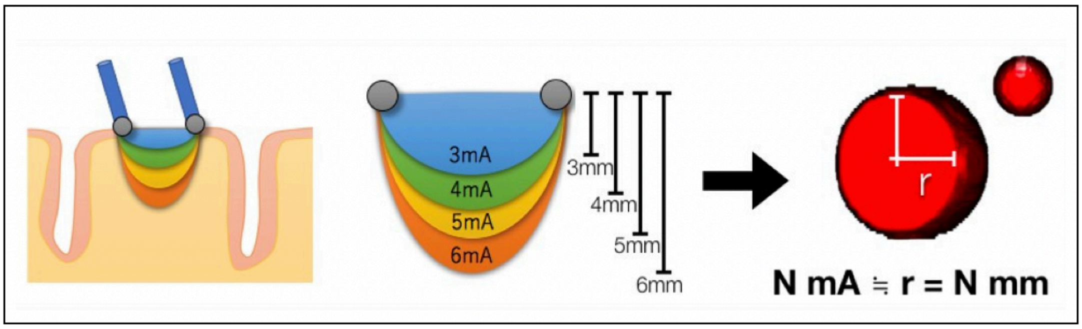

Supplementary Figure 2. The relationship between intensity of the stimulation current and the depth of the stimulation point.

1-6mA of stimulation intensity corresponds 1-6mm distance in direct proportion. We can confirm the electric charging depth using proportional volume of the positive mapping point according to the correspondence.

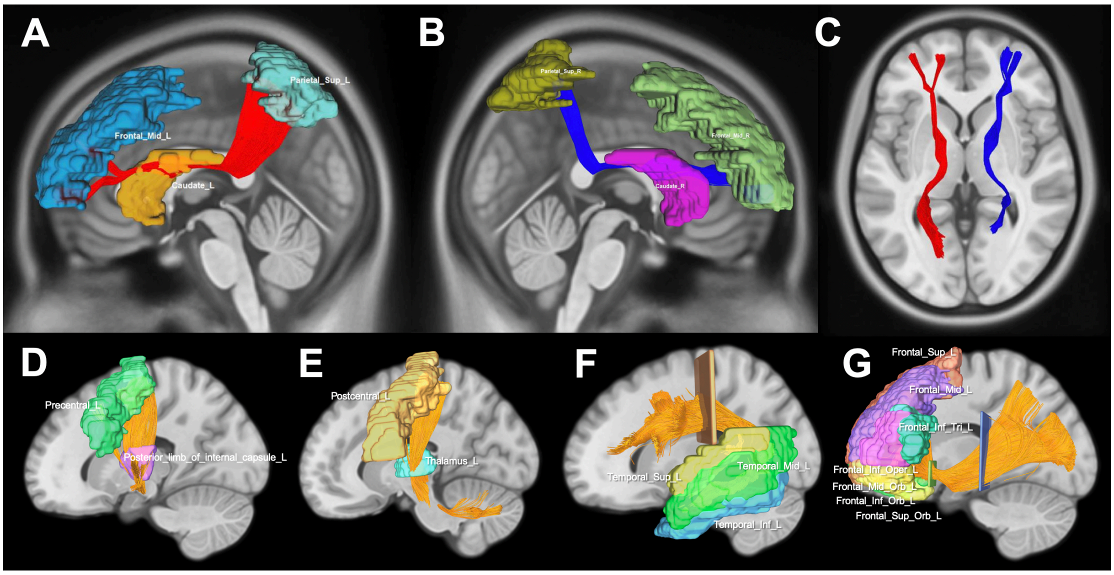

Supplementary Figure 3. Reconstruction of the Superior fronto-occipital fasciculus (SFOF) and other white matter fascicles.

A. The SFOF (red) on the left hemisphere. Regions of Interest (ROIs) are middle frontal gyrus (blue) and superior parietal gyrus (cyan). And caudate nucleus (orange) is set as seed in the DSI Studio. Frontal_Mid_L, left middle frontal gyrus; Caudate_L, left caudate nucleus; Parietal_Sup_L, left superior parietal gyrus.

B. The SFOF (blue) on the right hemisphere. Frontal_Mid_R, right middle frontal gyrus; Caudate_R, right caudate nucleus; Parietal_Sup_R, right superior parietal gyrus.

C. The SFOF (red) of the left hemisphere and the SFOF (blue) of the right hemisphere on the axial section.

D. Pyramidal tract (orange) of the left hemisphere. ROIs are the precentral gyrus (green) and posterior limb of internal capsule (pink). Precental_L, left precentral gyrus; Posterior_limb_of internal_capsule_L, left posterior limb of internal capsule.

E. Somatosensory tract (orange) of the left hemisphere. ROIs are the postcentral gyrus (yellow) and thalamus (cyan). Postcentral_L, postcentral gyrus; Thalamus_L; left thalamus.

F. Arcuate fascicle (orange) of the left hemisphere. Seeds are superior (yellow), middle (green) and inferior temporal giri (blue), and a ROI (ivory) is drawn in the coronal plane of left frontoparietal area. Temporal Inf_L, Inferior temporal gyrus;

Temporal_Mid_L, left middle temporal gyrus; Temporal_sup_L, left superior temporal gyrus.

G. Inferior fronto-occipital fascicle (orange) of the left hemisphere. Seeds are superior frontal gyrus including the orbital part (green), middle frontal gyrus including the orbital part (skin color), inferior frontal gyrus including the orbital (yellow), triangular (purple) and opercular parts (cyan). Two ROI masks (blue and yellow green) were drawn to select only the fibers that passed from the frontal lobe to the posterior region. Frontal_Inf_Oper_L, left opercular part of inferior frontal gyrus; Frontal_Inf_Orb_L, left orbital part of inferior frontal gyrus; Frontal_Inf_Tri_L, left triangular part of inferior frontal gyrus; Frontal_Mid_L, left middle frontal gyrus; Frontal_Mid_Orb_L, left orbital part of middle frontal gyrus; Frontal_Sup_L, left superior frontal gyrus; Frontal_Sup_Orb_L, left orbital part of superior frontal gyrus.

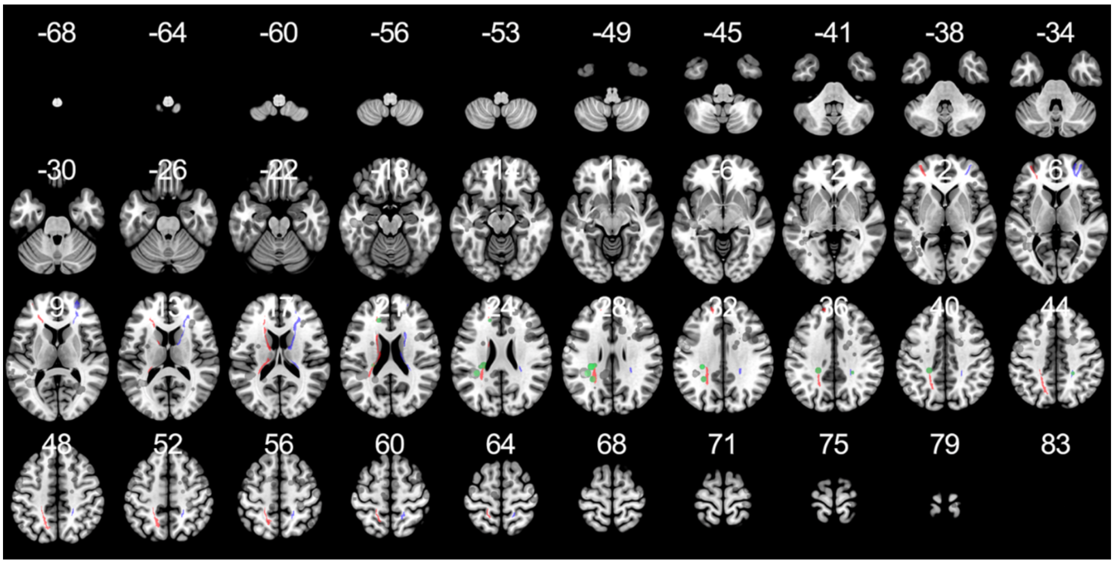

Supplementary Figure 4. Positive mapping points in anatomical relationships with superior fronto-occipital fascicle on a normal brain template

All 453 positive mapping points (gray) and eight positive sites (green) on the superior fronto-occipital fascicle reconstructed by diffusion spectrum imaging (left, red; right, blue) are visualized in axial images with Montreal Neurological Institute (MNI) coordinates.

Supplementary material 1

Fiber dissection of the acallosal brain

Objectives

In this supplementary material, our purpose is to demonstrate the differences between the acallosal brain case and normal brain.

Materials and methods

12 normal postmortem hemispheres were used as the control for comparison with the acallosal brain. Operations were approved by the ethics committee at Kanazawa University.

The same fiber dissection method of the SFOF study was used to dissect the hemispheres.

Results

The clinical remarks and surface anatomy of the acallosal brain

The donor was 82 years old and right-handed man. He had received a higher education and lived without any social and vocational difficulties. He was diagnosed as an early stage of stomach cancer at the age of 40 years old and recovered completely. He was hospitalized due to pneumonia for 19 days until his death. There was no episode of mental and psychological abnormality throughout his life.

The disposition of the sulci and gyri on the lateral surface of the control brain (Figs. 1A and B) and acallosal brain (Fig. 1C and D) did not remarkably differ. However, the medial surface of the acallosal brain (Fig. 2C and D) had several obvious differences from the control brain (Fig. 2A and B); (1) the rostrum, genu and anterior trunk were extraordinary thick to bury the anterior horn of the lateral ventricle, forming a Probst bundle-like white matter, (2) the posterior half of the corpus callosum became attenuated and the splenium was lacking, (3) the septum pellucidum was vestigial, (4) the isthmus of the cingulate gyrus was lacking, (5) two gyri in the precuneus extended

into the area of the splenium, (6) the parahippocampal gyrus and uncus enlarged remarkably, (7) the anterior commissure was approximately 4 mm in diameter.
The surface structures of the right and left hemispheres in the acallosal brain were not different from each other, as shown in Figure 1 and 2.

The results of fiber dissection in the control and acallosal brains

The results of the right hemisphere were presented here, since the dissection was more precisely documented in this side.

In the control hemisphere (Fig. 3A), the corpus callosum fibers were carefully nipped off with a fine forceps and the spaciousness of the anterior horn of the lateral ventricle was examined. The ependyma (E) covered the inferior surface of the corpus callosum (CC/E) and continuously wrapped over the superior surface of the caudate nucleus and thalamus (CN/E and T/E).

In the acallosal hemisphere, the corpus callosum and fornix made a mass of fibers (Probst bundle, PB) with variety of orientation, which was large enough to bury almost the entire anterior horn of the lateral ventricle (Fig. 2C and D). The mass was removed by, firstly, dividing it into small fiber bundles and then they were nipped off with forceps (Fig. 3C). The direction of the fibers was variable and not always rectangular to the sagittal plane. They tended to change from antero-medial to postero-lateral, especially, near the fornix. Since we realized the partial callosal agenesis only after division of the brain into the right and left hemispheres, we were not able to confirm the presence of the frontal sigmoid bundle. This mass of fibers (the remaining fibers after removing, PB) rested on a thin layer of the ependyma (E). The ependymal layer was discontinuous along the lamina affixa on the thalamus (arrows), which appeared arbitrary dividing into the antero-superior callosal fiber area (E) and postero-inferior fornix fiber area. The fornix was traceable from the column that formed, as usual, the anterior wall of the interventricular foramen to the peduncule of the fornix that fanned out posteriorly, continuing to the fimbria of the hippocampus.

After removal of the antero-superior part of the ependyma (Fig. 3D), the remaining

ependyma covered the caudate nucleus (CN/E), stria terminalis and lamina affixa attached by the choroid plexus. The corpus callosum fibers which have been pinched off shortly (CC) continued far backward to where, in the control brain, the splenium should be located.

In the control brain (Fig. 3B), the cingulate gyrus (CG) continued to the isthmic gyrus which further continued to the parahippocampal gyrus and, thus, the isthmic gyrus (I) white matter continued to the parahippocampal white matter (an arrow).

In the acallosal brain (Fig. 3E~H), the cingulate gyrus continued posteriorly with an extension of the aberrant precuneus gyrus (Fig. 3E and F, arrows) and the extension connected to an aberrant short gyrus (Fig. 3G, black asterisk). The short gyrus (Fig. 3F, white asterisk) was located more deeply (=laterally) than the normal isthmic gyrus and continuous with the parahippocampal gyrus (Fig. 3E and F). The parahippocampal gyrus joined with the lingual gyrus (Fig. 3H, arrow) as usual manner.

Supplementary material 1

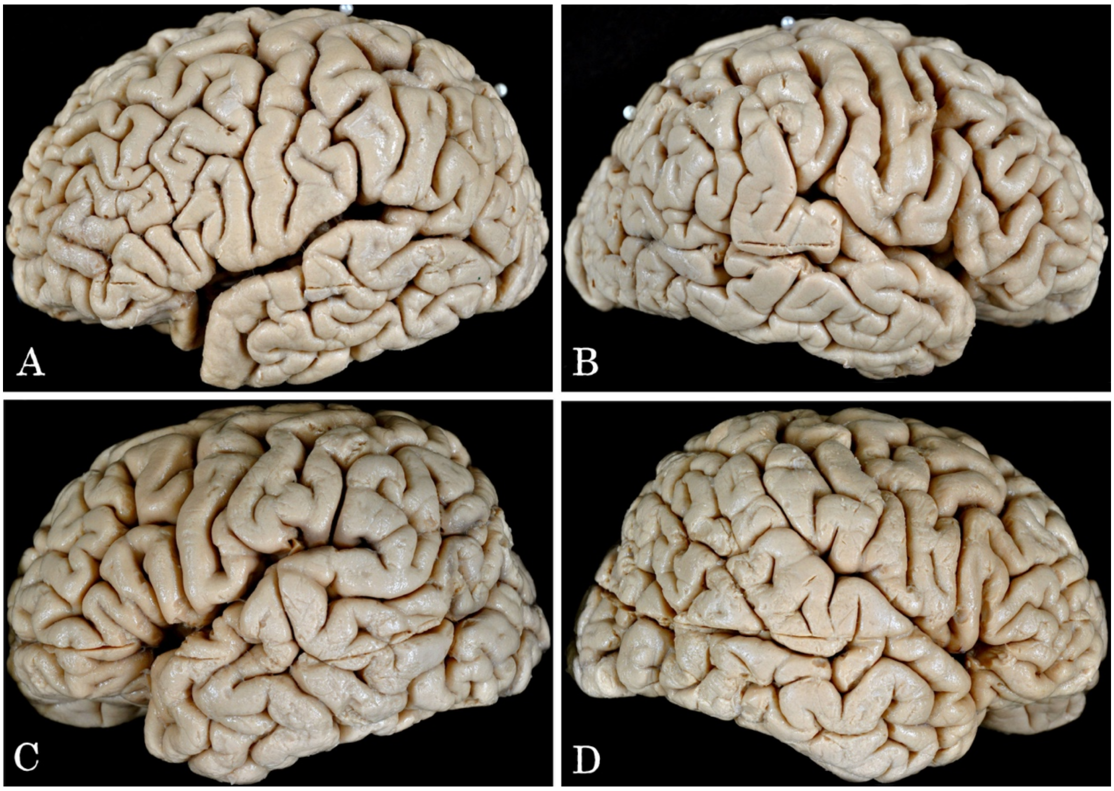

Figure 1. The lateral surface of the control (A, B) and acallosal (C, D) hemispheres. No remarkable difference was found between the control and acallosal brains. The anterior white ball indicated the central sulcus, and the posterior white ball indicated the parieto-occipital sulcus. The color balls were 4.0 mm in diameter and utilized as a scale, too.

Supplementary material 1

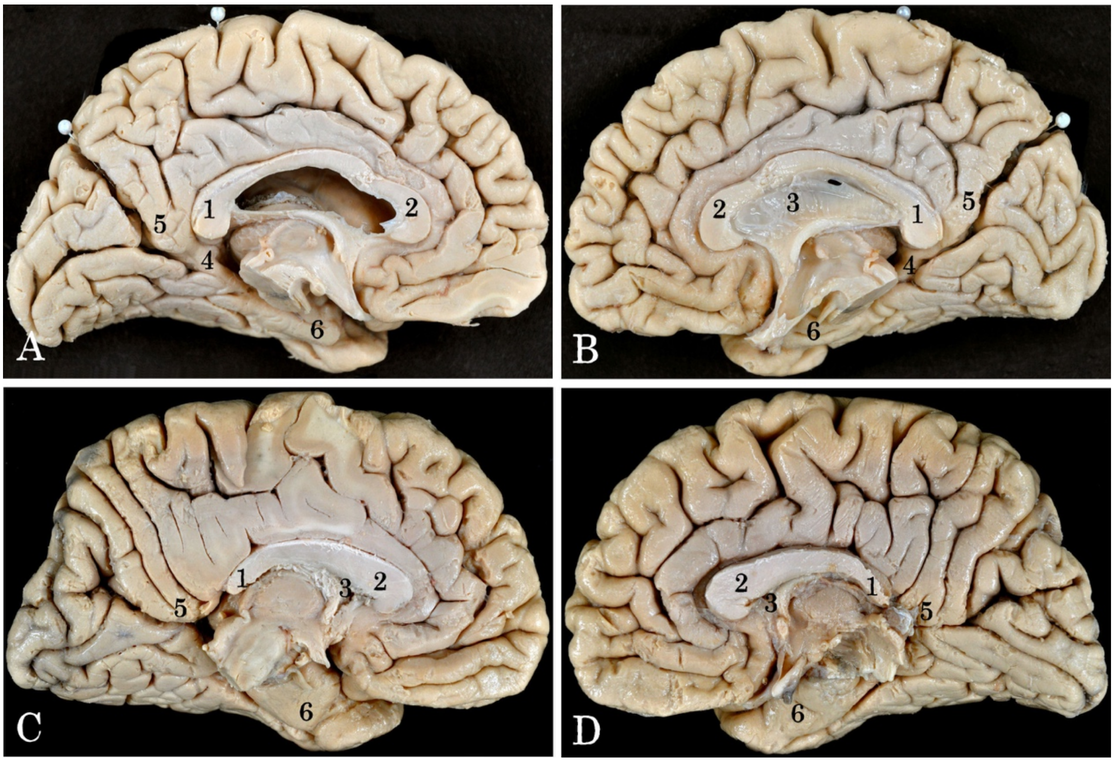

Figure 2. The medial surface of the control (A, B) and acallosal (C, D) hemispheres.

There were seven structural differences (numbered 1 to 7) in the acallosal brain. (1) the posterior half of the corpus callosum became attenuated and the splenium was lacking, (2) the rostrum, genu and anterior trunk were extraordinary thick to bury the anterior horn of the lateral ventricle, forming a Probst bundle-like white matter, (3) the septum pellucidum was vestigial, (4) the isthmus of the cingulate gyrus was lacking, (5) two gyri in the precuneus extended into the area of the splenium, (6) the parahippocampal gyrus and uncus enlarged remarkably, (7) the anterior commissure was approximately 4 mm in diameter.

Supplementary material 1

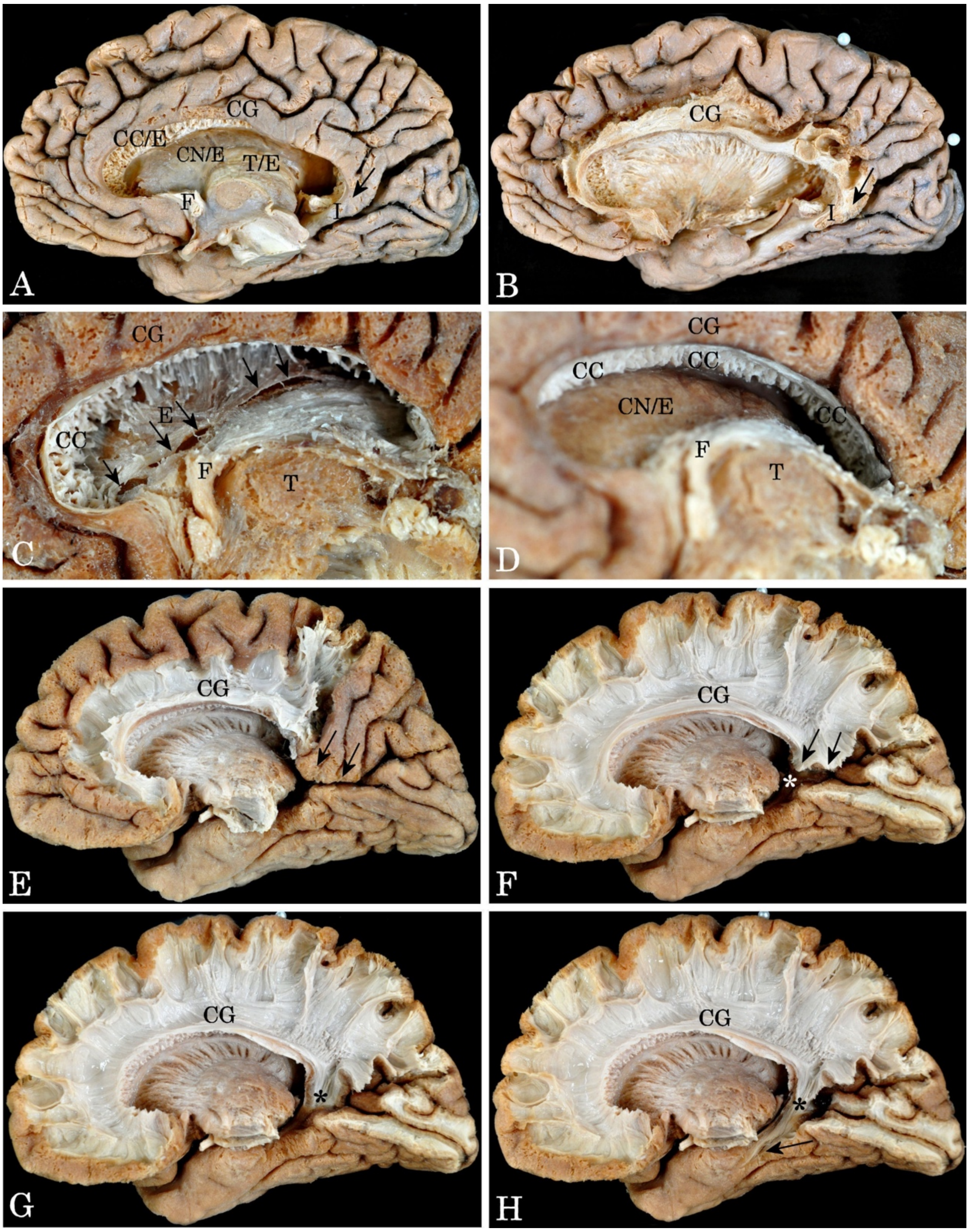

Figure 3. The medial structures of the control (Fig. A and B) and acallosal (Fig. C-H) hemispheres.

In the control cerebral hemisphere (Fig. A), the ependyma covered the inferior surface of the corpus callosum, continued inferiorly, and covered over the medial surface of the caudate nucleus and thalamus. In the acallosal hemisphere (Fig. C), the thickened corpus callosum pushed down the ependyma and crus of the fornix to the

thalamic surface. The ependyma was discontinuous (Fig. C, arrows) along the lamina affixa to which the choroid plexus attached. By the removal of the ependyma along the lamina affixa (Fig. D), it became clear that the inferiorly folded ependyma covered the caudate nucleus, as did in the control hemisphere. The fornix ran backward forming the interventricular foramen (Fig. D), with aberrant callosal fibers, on the thalamic surface and continued to the fimbria of the hippocampus.

In the control cerebral hemisphere, the cingulate gyrus (Fig. B, CG) continued to the isthmic gyrus that further continued to the parahippocampal gyrus (Fig. B, arrows). In the acallosal brain, the cingulum white matter (Fig. E, arrow) continued posteriorly with an extension of the aberrant precuneus gyrus and the extension connected to an aberrant short gyrus (Fig. F, asterisk). The short gyrus was located more deeply (=laterally) than the normal isthmic gyrus and continuous to the parahippocampal gyrus from the more lateral side (Fig. G and H).
